# Intravitreal vascular endothelial growth factor inhibitors did not increase the risk of end-stage renal disease in patients with biopsy-proven diabetic kidney disease based on matched study

**DOI:** 10.3389/fphar.2022.1077047

**Published:** 2022-12-07

**Authors:** Xiang Xiao, Junlin Zhang, Shuming Ji, Yutong Zou, Yucheng Wu, Chunmei Qin, Jia Yang, Yuancheng Zhao, Qin Yang, Fang Liu

**Affiliations:** ^1^ Division of Nephrology, West China Hospital of Sichuan University, Chengdu, China; ^2^ Division of Nephrology, The First Affiliated Hospital of Chendu Medical College, Chengdu, China; ^3^ Laboratory of Diabetic Kidney Disease, Centre of Diabetes and Metabolism Research, West China Hospital of Sichuan University, Chengdu, China; ^4^ Division of Project Design and Statistics, West China Hospital of Sichuan University, Chengdu, China

**Keywords:** aflibercept, Bevacizumab, ranibizumab, diabetic kidney disease, end-stage renal disease

## Abstract

**Purpose:** This study aimed to investigate the effects of intravitreal (IVT) VEGFi on long-term renal outcomes in patients with biopsy-proven diabetic kidney disease (DKD).

**Patients and methods:** Patients prescribed IVT VEGFi (VEGFi group) were enrolled from a retrospective cohort with biopsy-proven DKD, and those not prescribed VEGFi (non-VEGFi group) were enrolled by 1:3 propensity score matching, adjusted for clinical and pathological baseline indicators. The primary endpoint is defined as end-stage renal disease (ESRD) and the secondary endpoint is defined as all-cause mortality.

**Results:** Compared with patients in non-VEGFi group, patients with VEGFi had a higher proportion of diabetic retinopathy (DR) (50.9% vs 100%, *p* < 0.001) before matching. Standardized mean difference (SMD) of age, DR, duration of diabetes, the proportion of hypertension, eGFR, initial proteinuria, serum albumin, hemoglobin, the proportion of RAAS inhibitor and interstitial fibrosis and tubular atrophy (IFTA) were >10%. After matching, there was no significant difference in clinical pathology between the two groups. Except for the proportion of hypertension, the SMD of other indicators was <10%. Endpoints such as ESRD (Log-Rank *p* = 0.772) and all-cause mortality (Log-Rank *p* = 0.834) were not significantly different between the two groups.

**Conclusion:** Our data suggested that IVT VEGFi did not increase the incidence of ESRD and all-cause mortality in patients with DKD.

## Introduction

Diabetic kidney disease (DKD) and diabetic retinopathy (DR) are microvascular complications of diabetes mellitus (DM), and approximately 50%–60% of patients with type 2 diabetes mellitus (T2DM) and DKD have combined DR (Penno et al., 2012). In recent years, intravitreal (IVT) vascular endothelial growth factor inhibitors (VEGFi) have gradually become a first-line treatment option for DR (Schmidinger, 2013). The three most commonly used anti-VEGF agents include bevacizumab, ranibizumab, and aflibercept. Their initial primary application was as an intravenous chemotherapeutic adjunct in the form of bevacizumab, for the treatment of solid tumors, including breast, colorectal, and lung cancer (Jain et al., 2006).

Vascular endothelial growth factor (VEGF) is a key factor in the formation of diabetic retinopathy (DR), and patients with DR have elevated expression of VEGF and receptors, and IVT injection of VEGFi drugs can promote regression of neovascularization, attenuate leakage, reduce neovascularization, and effectively treat retinopathy (Wells et al., 2015). It is widely used for proliferative diabetic retinopathy (PDR), diabetic macular edema (DME), and as a preoperative medication for DR vitreous surgery (Korobelnik et al., 2014; (Apte et al., 2007). IVT injection of VEGFi drugs does not increase the systemic risk due to local administration and the small dosage, however, the local drug still needs to be cleared by the circulatory system (Apte et al., 2007), which can lead to the VEGF system inhibition effect.

However, VEGFi also plays an important role in maintaining endothelial cell function including glomeruli and the peritubular capillary system, and the possible adverse effects of intravenous VEGFi therapy such as hypertension, proteinuria, and thrombotic events have attracted increasing attention (Eremina et al., 2008; (Pellé et al., 2011; (Hayman et al., 2012). Diabetic patients present with DR more often with DKD (He et al., 2013) and several cardiovascular complication (Papatheodorou et al., 2016), so the potential risks of IVT VEGFi therapy in DR patients such as proteinuria, hypertension, and kidney injury need to be concerned.

However, the conclusions of current studies on the renal effects of IVT administration of VEGFi are inconsistent. In addition, some of the patients in the study did not have biopsy-proven DKD, it cannot be excluded that the patient’s kidney injury was the result of other kidney diseases or a combination of others. Finally, previous studies focused more on short-term renal outcomes, such as the occurrence of AKI, changes in proteinuria, blood pressure, and eGFR, and lacked long-term observations, such as the effect on progression to end-stage renal disease (ESRD) and all-cause mortality. Our study aimed to discuss the effects of IVT administration of VEGFi on long-term renal outcomes in patients with DKD.

## Material and methods

### Study design and patients

**FIGURE 1:**
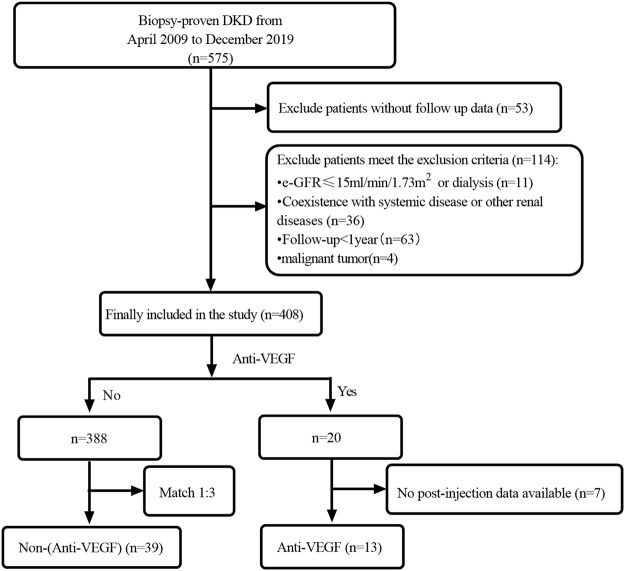
Flowchart of study participants.

This is a retrospective cohort study including T2DM patients with biopsy-confirmed DKD at the West China Hospital of Sichuan University from April 2009 to December 2019. The diagnosis and classification of T2DM were based on the criteria of the American Diabetes Association, 2017 Standards of medical care in diabetes-2017 abridged for primary care providers, 2017) and DKD was according to the standards of the Renal Pathology Society (RPS) in 2010 (Tervaert et al., 2010). The inclusion criteria were 1) age≥ 18 years old, 2) a diagnosis of T2DM, and 3) the diagnosis of DKD proven by renal biopsy. The exclusion criteria were 1) malignant tumor, 2) e-GFR<15ml/min/1.73 m^2^ or having been entrance into dialysis, 3) Follow-up time<1year, 4) with incomplete data, and 5) coexistence with other renal diseases (Figure 1). Patients who had filled a prescription for the IVT administration of VEGFi agent therapy (Conbercept, Ranibizumab, and Aflibercept) (VEGFi group) were screened from the included population. And then these patients were 1:3 propensity score matched (PSM) with patients who were not prescribed the VEGFi agent (non-VEGFi group) adjusting for gender, age, BMI, duration of diabetes, hypertension, initial proteinuria, e-GFR, serum albumin, hbA1c, hemoglobin, renin-angiotensin-aldosterone system (RAAS) inhibitor, Glomerular class, interstitial fibrosis and tubular atrophy (IFTA). This study has been approved by the ethics committee of West China Hospital of Sichuan University. The ethical committee clearance number was 2022 (1210). The study protocol complied with the ethical standards laid down in the 1964 Declaration of Helsinki and its later amendments. Written informed consent was obtained from all individual patients when hospitalized.

### 2 Clinical and pathologic characteristics

Clinical and pathologic information was collected from electronic medical records before a prescription for the IVT administration of VEGFi agent in the VEGFi group or at the time of renal biopsy in the non-VEGFi group. Subsequent follow-ups of these patients were performed 2–4 times per year based on the patient’s condition. The renal outcomes were defined by reaching ESRD, which was considered as the requirement of renal replacement therapy (RRT) (including hemodialysis or abdominal dialysis or kidney transplantation) or/and e-GFR<15ml/min/1.73 m^2^. The e-GFR was calculated by the creatinine-based Chronic Kidney Disease Epidemiology Collaboration equation (CKD-EPI). The histological lesions were evaluated according to the criteria of RPS (Tervaert et al., 2010).

### 3 Statistical analysis

The continuous variables were expressed as mean ± standard deviation (SD) or median with interquartile range (IQR), and categorical data were presented as frequency combined with percentages. Propensity scores were used to match each patient who initiated IVT VEGFi with patients who did not use VEGFi (in a 1:3 ratio) with a caliper of 0.1. After matching, a more than 10% standardized difference was used to detect a significant group imbalance between baseline variables (Normand et al., 2001). The Mann-Whitney U test was used for between-group tests of non-normal distribution, and the chi-square test for row × lists was used for between-group comparisons of categorical variables. Kaplan-Meier curves were generated to visualize the cumulative incidence of the renal outcome over time and log-rank tests were used to compare the survival distribution in the two groups. All statistical tests were analyzed by using SPSS version 26.0. Matching was done with the Match function in the R package Matching (R version 3.2.3). A two-sided *p*-value <0.05 was considered statistically significant.

## Results

### Baseline characteristics

The baseline data of the enrolled 408 patients before the match are shown in Table 1. Compared with patients in non-VEGFi group, patients with IVT VEGFi had a higher proportion of diabetic retinopathy (DR) (50.9% vs 100%, *p* < 0.001). Although the remaining indicators did not present significant statistical differences, standardized mean difference (SMD) of age, DR, duration of diabetes, the proportion of hypertension, eGFR, initial proteinuria, serum albumin, hemoglobin, the proportion of RAAS inhibitor, and interstitial fibrosis and tubular atrophy (IFTA) were >10%. To ensure greater consistency in baseline characteristics between groups, we performed propensity score matching 1:3. We matched patients for age, sex, BMI, the proportion of DR, duration of diabetes, the proportion of hypertension, eGFR, initial proteinuria, serum albumin, hemoglobin, glomerular class, IFTA, the proportion with RAAS using. A total of 52 patients were included after matching, including 13 in VEGFi group and 39 in non-VEGFi group (Figure 1). The baseline data of the enrolled 52 patients after the match are shown in Table 2. After matching, there were no significant statistical differences in all clinicopathological characteristics. Except for the proportion of hypertension, the SMD of clinicopathological characteristics between the two groups was< 10% in both groups (Table 2).

**TABLE 1:** Baseline clinicopathological characteristics of enrolled patients before the match.

Variables	All (*n* = 401)	Non-VEGFi (*n* = 388)	VEGFi (*n* = 13)	p-value	SMD
Gender (male, %)	285 (71.1)	276 (71.1)	9 (69.2)	0.882	0.041
Age (years)	51 (46–57)	51 (46–57)	51 (44–53)	0.342	0.311
BMI (kg/m^2^)	25.68 (23.24–27.54)	25.71 (23.35–27.57)	24.51 (21.29–26.53)	0.201	0.072
DR [n (%)]	204 (50.9)	201 (51.8)	13 (100)	<0.001	1.091
Duration of diabetes (Months)	96 (36–138)	96 (36–132)	156 (31–180)	0.288	0.262
Hypertension [n (%)]	346 (86.3)	337 (86.9)	9 (69.2)	0.069	0.424
e-GFR (ml/min/1.73m^2^)	60.15 (42.8–90.32)	60.14 (42.94–90.37)	61.50 (38.65–87.32)	0.749	0.162
Initial proteinuria (g/day)	3.95 (1.9–7.55)	3.96 (1.84–3.71)	3.74 (2.35–6.44)	0.971	0.208
Serum albumin (g/L)	34.60 (28.15–40.20)	34.7 (28.13–40.20)	32.60 (28.00–38.45)	0.568	0.119
Hemoglobin (g/L)	117.00 (102.00–117.00)	117.00 (102.25–136.75)	118.00 (87.00–138.00)	0.442	0.263
HbA1c (%)	7.30 (6.30–8.60)	7.30 (6.30–8.60)	6.35 (4.66–6.90)	0.905	0.028
RAAS inhibitor [n (%)]	320 (79.8)	312 (80.4)	8 (61.5)	0.095	0.415
Glomerular class [n (%)]				0.941	0.047
Ⅰ	19 (4.7)	19 (4.9)	0 (0.0)		
Ⅱa	80 (20.0)	77 (19.8)	3 (23.1)		
Ⅱb	51 (12.7)	49 (12.6)	2 (15.4)		
Ⅲ	188 (46.9)	182 (46.9)	6 (46.2)		
Ⅳ	63 (15.7)	61 (15.7)	2 (15.4)		
IFTA [n (%)]				0.615	0.361
0	10 (2.5)	10 (2.6)	0 (0.0)		
1	183 (45.6)	179 (46.1)	4 (30.8)		
2	166 (41.4)	159 (41.0)	7 (53.8)		
3	42 (10.5)	40 (10.3)	2 (15.4)		

DR, diabetic retinopathy; e-GFR, estimated glomerular filtration rate; RAAS, Renin-angiotensin-aldosterone System; IFTA, interstitial fibrosis and tubular atrophy; SMD, STD, mean difference.

**TABLE 2:** Baseline clinicopathological characteristics of enrolled patients after the match.

Variables	All (*n* = 52)	Non-VEGFi (*n* = 39)	VEGFi (*n* = 13)	p-value	SMD
Gender (male, %)	36 (69.2)	27 (69.2)	9 (69.2)	1.000	0.000
Age (years)	50 (42–55)	48 (42–55)	51 (44–53)	0.775	0.093
BMI (kg/m^2^)	25.48 (23.10–27.08)	25.48 (23.11–27.08)	24.51 (21.29–26.53)	0.358	0.039
DR [n (%)]	52 (100.0)	39 (100.0)	13 (100.0)	1.000	0.000
Duration of diabetes (Months)	96 (36–138)	120 (60–156)	156 (31–180)	0.575	0.042
Hypertension [n (%)]	34 (65.4)	25 (64.1)	9 (69.2)	0.736	0.106
e-GFR (ml/min/1.73m^2^)	60.21 (40.22–78.73)	54.82 (40.33–78.32)	61.50 (38.65–87.32)	0.759	0.024
Initial proteinuria (g/day)	2.95 (2.00–6.35)	2.83 (1.84–6.40)	3.74 (2.35–6.44)	0.460	0.000
Serum albumin (g/L)	33.55 (27.48–39.28)	34.30 (26.80–39.30)	32.60 (28.00–38.45)	0.916	0.049
Hemoglobin (g/L)	115.50 (93.00–128.75)	114.00 (94.00–126.00)	118.00 (87.00–138.00)	0.916	0.024
HbA1c (%)	6.70 (5.95–8.48)	6.60 (5.80–8.50)	6.90 (6.35–9.05)	0.472	0.073
RAAS inhibitor [n (%)]	32 (61.5)	24 (61.5)	8 (61.5)	1.000	0.000
Glomerular class [n (%)]				0.900	0.047
Ⅰ	1 (1.9)	1 (2.6)	0 (0.0)		
Ⅱa	12 (23.1)	9 (23.1)	3 (23.1)		
Ⅱb	5 (9.6)	3 (7.7)	2 (15.4)		
Ⅲ	24 (46.2)	18 (46.2)	6 (46.2)		
Ⅳ	10 (19.2)	8 (20.5)	2 (15.4)		
IFTA [n (%)]				0.812	0.072
0	0 (0.0)	0 (0.0)	0 (0.0)		
1	19 (36.5)	15 (38.5)	4 (30.8)		
2	24 (46.2)	17 (43.6)	7 (53.8)		
3	9 (17.3)	7 (17.9)	2 (15.4)		
Endpoint [n (%)]					
ESRD	31 (59.6)	26 (66.7)	6 (46.2)	0.188	
Death	3 (5.8)	2 (5.1)	1 (7.7)	0.731	

DR, diabetic retinopathy; e-GFR, estimated glomerular filtration rate; RAAS, Renin-angiotensin-aldosterone System; IFTA, interstitial fibrosis and tubular atrophy; SMD, STD, mean difference; ESRD, end-stage renal disease.

The median follow-up time was 30 months (17–52 months). The median time of DM duration was 96 months (36–138 months). The median eGFR was 60.21 ml/min/1.73 m^2^ (40.22–78.73 ml/min/1.73 m^2^). The median of proteinuria was 2.95 g/day (2.00–6.35 g/day). The pathologic characteristics showed that the degree of glomerular lesions was divided into Ⅰ, Ⅱa, Ⅱb, Ⅲ, and Ⅳ according to the glomerular class, accounting for 1.9% (1), 23.1% (12), 9.6% (5), 46.2% 24) and 19.2% (10), respectively. The proportion of patients using RAASi was 61.5% (32). The drug names and cumulative doses of all patients prescribed VEGFi were described in Table 3. The prescription proportions of aflibercept, concept, and ranibizumab were 15.4% (2), 46.1% (6), and 38.5% (5), respectively. The average cumulative doses of aflibercept, concept, and ranibizumab were 4 mg, 0.83 mg, and 0.8 mg, respectively.

**TABLE 3:** The VEGFi medication information of cases.

Number	Gender	Age (year)	Weight (kg)	Type of medication	Cumulative dose (mg)
1	Male	45	65	Conbercept	0.5
2	Male	50	66	Conbercept	0.5
3	Male	52	70	Conbercept	0.5
4	Male	34	60	Aflibercept	6
5	Male	38	78	Aflibercept	2
6	Female	59	56	Conbercept	1.5
7	Male	52	67	Conbercept	1.5
8	Female	33	65	Conbercept	0.5
9	Male	67	64	Ranibizumab	0.5
10	Female	51	58	Ranibizumab	1.5
11	Male	46	85	Ranibizumab	1
12	Male	55	73	Ranibizumab	0.5
13	Male	43	55	Ranibizumab	0.5

### Risks of progression to ESRD

**FIGURE 2:**
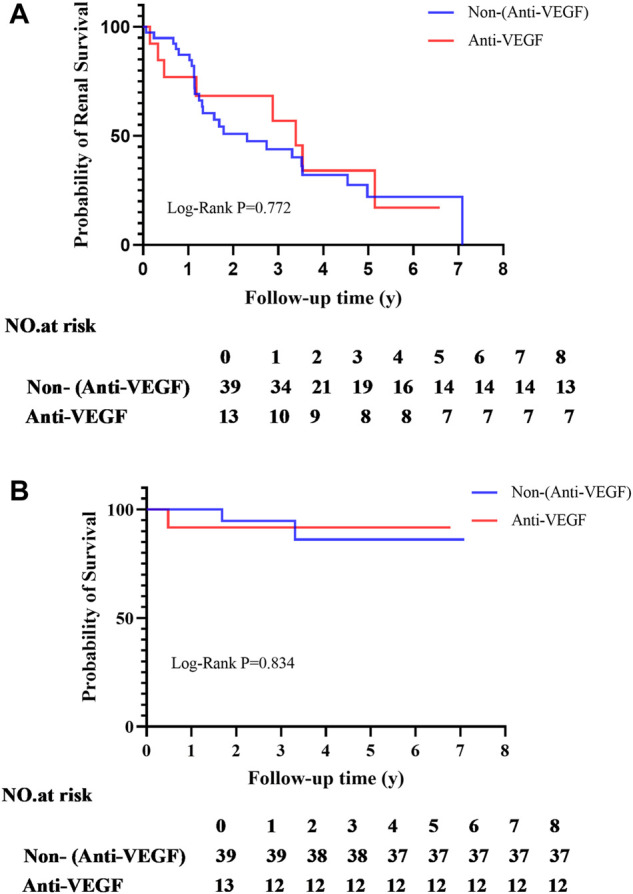
**(A)** Kaplan-Meier analyses of entering ESRD; **(B)** Kaplan-Meier analyses of survival rate.

During follow-up, 6 of 13 (46.2%) patients progressed to ESRD in the VEGFi group compared with 26 of 39 (66.7%) patients in the non-VEGFi group. The 1-year renal survival rates of VEGFi and non-VEGFi groups were 87.2% (34/39) and 76.9% (10/13), respectively. The 5-year renal survival rates of VEGFi and non-VEGFi groups were 53.8% (7/13) and 35.9% (14/39), respectively. The Kaplan–Meier analysis revealed that renal survival of patients after prescription of VEGFi is not deteriorated (Log-rank *p* = 0.772, Figure 2A).

During follow-up, 1 of 13 (7.7%) patient occurrence of death in the VEGFi group compared with 2 of 39 (5.1%) patients in the non-VEGFi group. The 1,5-year survival rates of VEGFi were 100% (39/39) and 94.9% (37/39), and non-VEGFi groupswere 92.3% (12/13) and 92.3% (12/13). The Kaplan–Meier analysis revealed that the survival of patients after prescription of VEGFi was not deteriorated (Log-rank *p* = 0.834, Figure 2B).

## Discussion

Our study provided data on long-term renal outcomes and all-cause mortality with VEGFi IVT use in patients with DKD, and the results suggested that the IVT use of VEGFi did not result in an increased risk of patients developing ESRD and survival.

The three most commonly used IVT VEGFi agents in ophthalmology include bevacizumab, ranibizumab, and aflibercept. Bevacizumab was also the first monoclonal anti-VEGF antibody used intravitreally to inhibit retinal neovascularization (Kazazi-Hyseni et al., 2010). The latest monoclonal VEGF inhibitor, ranibizumab, is a monoclonal κappa light chain antibody fragment, which was approved by FDA in 2012 (Hanna et al., 2019a). Ranibizumab is a shorter-acting agent (Hanna et al., 2019b) with efficacy comparable to that of bevacizumab (Azanza Perea and García Layana, 2012). Aflibercept is a VEGF trap for the soluble VEGF enzyme that was approved by the FDA in 2011. It is a dimer fused to the constant region Fc of human IgG1 and is fused to chimeric binding sites with sequences from VEGF receptors 1 and 2 (Hanna et al., 2019a). Three VEGFi were prescribed in our study, including conbercept (Chengdu Kang Hong biotechnologies Co. Ltd., China), ranibizumab (Novartis Pharma Stein AG, Schweiz), aflibercept (Bayer AG, Germany). So, regrettably, we did not have patients prescribed bevacizumab.

FDA data show detectable serum levels of ∼0.2 nmol/L for aflibercept Aflibercept [fda package insert] (Regeneron, 2011), and ∼0.05 nmol/L for ranibizumab Bevacizumab [fda package insert], (Genentech, 2004), and they suggest that these levels are ∼200-fold lower than the minimum concentrations required to maximally inhibit systemic VEGF. However, studies by Avery et al. (2014) showed that the level of IVT systemic absorption was greater and more significant than previously thought. The 50% inhibitory concentration (IC50) of anti-VEGF drugs approximated the systemically measured serum drug levels after the injection of standard doses (Avery et al., 2014; (Avery et al., 2017). The mean systemic levels after IVT aflibercept are well above the IC50 for several weeks (up to 30 days) (Avery et al., 2014; (Avery et al., 2017). Bevacizumab, systemic drug concentrations were closer to the IC50 and decreased below this level after 2 weeks of injection (Avery et al., 2014; (Avery et al., 2017). The initial post-injection concentrations of ranibizumab are near the IC50 but rapidly decrease to below the IC50 (in days) (Avery et al., 2014; (Avery et al., 2017). Multiple injections of VEGFi, systemic levels of these drugs (approaching the IC50 in some cases) can be detected in serum for up to 3 months (Avery et al., 2014; (Avery et al., 2017). More recently, Jampol et al. and Hirano et al. showed that aflibercept and bevacizumab could cause inhibition of systemic VEGF for>4 weeks after IVT injection (Hirano et al., 2018; (Jampol et al., 2018). There was not a similar decrease in systemic VEGF levels with ranibizumab (Jampol et al., 2018). Through these above studies, it is illustrated that intraretinal injection of VEGFi may lead to the occurrence of nephrotoxicity. Because the drugs are expensive and patients need to use these drugs at their own expense, it is difficult for drugs to be widely used, our patients have only used VEGFi1-2 times, but even a single use can lead to an increase in the systemic concentration of VEGFi in patients through the previous studies we listed. Therefore, it is necessary for us to explore further the impact of VEGFi on the prognosis of DKD patients.

Animal study (Tschulakow et al., 2014) found that after IVT aflibercept injection, the drug could deposit in the glomerulus and reduce the local VEGF level in the glomerulus. From the currently available reports in humans, the main kidney injuries attributed to VEGFi include hypertension, new onset proteinuria or proteinuria deterioration, eGFR decline, development of secondary glomerulonephritis, the appearance of TMA, and progression to ESRD. Several studies have reported that intraretinal injection of VEGFi leads to an increase in blood pressure. In a case series that included three patients, it was found that prescribing VEGFi can lead to the aggravation of hypertension in addition to an increase in proteinuria (Hanna et al., 2019b). A prospective observational study performed on 40 patients with DKD found that 1 month after bevacizumab injection, diastolic blood pressure and Hb level might be increased in patients, but deterioration of renal function and increase of proteinuria was not observed (Bagheri et al., 2018). However, the patients included in these studies could not be excluded from having comorbid non-diabetic kidney disease. Some cases reported that VEGFi could lead to glomerulonephritis. A case reported recurrence of minimal change nephropathy after IVT bevacizumab for 9 days (Sato et al., 2013), and a similar case was reported by Pérez-Valdivia et al. (Pérez-Valdivia et al., 2014). However, we know that minimal change nephropathy itself is prone to recurrence, and it is difficult to clarify whether these patients have other predisposing factors for recurrence. Cheungpasitporn et al. (2015) reported renal injury after intraretinal VEGFi in two series of renal transplant patients, one patient presenting with phospholipase A2 receptor-negative membranous nephropathy and one patient presenting with increased proteinuria, and they speculated that VEGFi maybe a role in modulating antibody-mediated phenomena. There were also many studies reported that intraretinal VEGFi could lead to the occurrence of thrombotic microangiopathy (TMA). Pellé et al. (2011) reported the first patient with an acute decline in renal function, non-immune microangiopathic hemolytic anemia with schistocytes and thrombocytopenia after IVT ranibizumab, and light microscopy of a kidney biopsy specimen showed segmental duplication of the glomerular basement membrane with endothelial swelling and multiple recanalized arteriolar thrombi. A recent study reported three TMA cases likely associated with IVT anti-VEGF therapy (Hanna et al., 2020). Phadke et al. (2021) recently also reported a TMA case with collapsing focal segmental glomerulosclerosis (cFSGS) in the setting of VEGFi administration. Besides, most reports are about the decrease of eGFR, and the deterioration of proteinuria. A retrospective study included 69 patients, and the final results suggested no change in eGFR after intraretinal VEGFi for 7–30 days (Kameda et al., 2018). A Planned retrospective analysis of randomized trial enrolled 660 patients and the final conclusion was that the patients who used of VEGFi had no long-term change in HTN or category of albuminuria (Glassman et al., 2018).

In addition, there are some studies on the risk of death in patients treated with IVT VEGFi, and these findings are inconsistent. A retrospective case-control study reported that IVT injection of VEGFi could increase all-cause mortality. After adjusting for confounding factors, Cox survival regression analysis showed that the death risk of patients with IVT injection of VEGFi was 1.69 times higher than that of the control group (Hanhart et al., 2017). The team also analyzed the impact of VEGFi on the mortality rate after acute myocardial infarction. The results showed that patients who injected VEGFi into the retina had an increased risk of death, and the shorter the time interval between VEGFi and acute myocardial infarction, the higher the risk of death (Hanhart et al., 2018a). They also found increased mortality within 3 months after a cerebrovascular event in patients treated with bevacizumab (Hanhart et al., 2018b). Although the above results suggested that the effects of VEGFi on patients may be long-term, the reason why these results appear was still unclear. However, Dalvin et al. (2019) obtained the opposite results, this retrospective cohort study assessed the risk of stroke, myocardial infarction, and death after IVT VEGFi for 5 years in patients using Kaplan Meier and Cox proportional hazards regression models, and it was not associated with an increased risk of stroke, myocardial infarction, or death. So, we need more studies to clarify whether VEGFi has long-term side effects.

Long-term prognosis, in general, is important, especially for ESRD. Our study was the first to investigate renal outcomes after intraretinal VEGFi in a cohort by matching clinical and pathological characteristics. The patients in our study were all patients with DKD confirmed by biopsy and were not complicated by other renal diseases. The results of our study confirmed that the use of VEGFi in patients with DKD has no significant effect on the long-term renal outcomes of the patients. So, in a long run, the use of VEGFi in patients with DR is relatively safe. However, it is best to take measures to avoid the occurrence of nephrotoxicity (Hanna et al., 2019a).

Of course, there are some limitations to this study. Firstly, our study was a retrospective study, so there were some inevitable selection biases. Meanwhile, the sample size was small. Finally, the proportion of hypertension did not match perfectly. However, in view that our study matched a very large number of clinicopathological indicators, and that many indicators were continuous numerical variables, the results of the matching have been excellent.

## Data Availability

The raw data supporting the conclusion of this article will be made available by the authors, without undue reservation.
